# Neuropsychological Evaluation and Functional Magnetic Resonance Imaging Tasks in the Preoperative Assessment of Patients with Brain Tumors: A Systematic Review

**DOI:** 10.3390/brainsci13101380

**Published:** 2023-09-28

**Authors:** Marta Pertichetti, Daniele Corbo, Francesco Belotti, Francesca Saviola, Roberto Gasparotti, Marco Maria Fontanella, Pier Paolo Panciani

**Affiliations:** 1 Neurosurgery Unit, Department of Medical and Surgical Specialties, Radiological Sciences and Public Health, University of Brescia and ASST Spedali Civili Hospital, 25123 Brescia, Italymarco.fontanella@unibs.it (M.M.F.); pierpaolo.panciani@unibs.it (P.P.P.); 2 Department of Medical and Surgical Specialties, Radiological Sciences and Public Health, University of Brescia, 25123 Brescia, Italy; daniele.corbo@unibs.it (D.C.); francesca.saviola@unibs.it (F.S.); roberto.gasparotti@unibs.it (R.G.); 3 Neuroradiology Unit, ASST Spedali Civili of Brescia, 25123 Brescia, Italy

**Keywords:** glioma, brain tumor, surgery, fMRI, neuropsychological evaluation

## Abstract

Background: Current surgical treatment of gliomas relies on a function-preserving, maximally safe resection approach. Functional Magnetic Resonance Imaging (fMRI) is a widely employed technology for this purpose. A preoperative neuropsychological evaluation should accompany this exam. However, only a few studies have reported both neuropsychological tests and fMRI tasks for preoperative planning—the current study aimed to systematically review the scientific literature on the topic. Methods: PRISMA guidelines were followed. We included studies that reported both neuropsychological tests and fMRI. Exclusion criteria were: no brain tumors, underage patients, no preoperative assessment, resting-state fMRI only, or healthy sample population/preclinical studies. Results: We identified 123 papers, but only 15 articles were included. Eight articles focused on language; three evaluated cognitive performance; single papers studied sensorimotor cortex, prefrontal functions, insular cortex, and cerebellar activation. Two qualitative studies focused on visuomotor function and language. According to some authors, there was a strong correlation between performance in presurgical neuropsychological tests and fMRI. Several papers suggested that selecting well-adjusted and individualized neuropsychological tasks may enable the development of personalized and more efficient protocols. The fMRI findings may also help identify plasticity phenomena to avoid unintentional damage during neurosurgery. Conclusions: Most studies have focused on language, the most commonly evaluated cognitive function. The correlation between neuropsychological and fMRI results suggests that altered functions during the neuropsychological assessment may help identify patients who could benefit from an fMRI and, possibly, functions that should be tested. Neuropsychological evaluation and fMRI have complementary roles in the preoperative assessment.

## 1. Introduction

Patients diagnosed with brain tumors may have variable prognoses influenced by histology and the molecular profile of the neoplastic formation, and by the degree of resection achieved in the tumor during surgery [1,2,3]. Indeed, among the several prognostic factors suggested in the literature, the extent of resection (EOR) based on objective tumoral volume analysis seems to be one of the main predictors of overall survival [4,5,6,7]. Surgical treatment, however, can only rarely be considered radical due to the infiltrating nature of gliomas per se [2]. Therefore, in recent years, treatment paradigms shifted from surgery focused on gross-total resection (GTR) to a maximally safe and function-preserving resection approach [8,9,10,11]. This transition was possible thanks to changes in the clinical approach by applying technological and conceptual innovations to improve safety during surgery, such as intraoperative ultrasound, cortical mapping, awake surgery, and tumor margin detection with fluorescence dye [4,5,12,13,14]. Parallel to this, the preoperative evaluation of patients and accurate presurgical planning also improved, including different noninvasive methods to identify the relationship between the brain tumor and eloquent areas, both at the cortical and subcortical levels [15].

Firstly, Functional Magnetic Resonance Imaging (fMRI) started to be widely employed for this purpose [16,17], allowing the creation of a functional map of the eloquent brain regions based on modifications in blood oxygenation levels by using the blood oxygen level-dependent (BOLD) contrast [18,19]. Specifically, fMRI can be employed at rest while the subject is lying still in the scanner and instructed to think about nothing in particular. In this case, fMRI measures the inter-regional dependencies across the brain and can be applied in presurgical functional mapping [20]. Otherwise, fMRI can be employed during the execution of a cognitive task (i.e., task-based fMRI). Task-based fMRI compares BOLD signal changes while performing specific tasks to baseline conditions, assuming that increased cerebral blood flow reflects neuronal activity [21]. Among several potential tasks suitable for fMRI experiments, a defined group of tasks are commonly applied for presurgical mapping in gliomas: sensorimotor, language-related, and executive function tasks [19,22,23]. In this context, the sensorimotor paradigm demonstrated high reliability [19,24,25], whereas mapping language and other higher cognitive functions is more debated regarding both the anatomical specificity and the paradigm’s sensitivity [18,19,26]. Paradigms for identifying visual and somatosensory areas have also been proposed.

Secondly, the neuropsychological evaluation is another important aspect regarding the preoperative assessment of patients with brain tumors [27,28]. Patients can develop impairments in multiple cognitive domains before or after surgery [29]. Therefore, the evaluation of cognitive function is essential for informing management and monitoring the long-term effects of tumors [29]. However, the wide range of existing tests reflects the fractionation of the cognitive system, and an in-depth assessment can take several hours. Furthermore, the wide range and variety of available tests may lead to reduced overlap between those used from one center to another, making the comparison of outcomes complex [29]. The presurgical combination of fMRI and neuropsychological assessment should help define (i) the tumor’s anatomical features, such as tumor site compared to fMRI-positive areas, plasticity phenomena, and prediction of EOR; (ii) the tumor’s functional effects on cognition. These data not only inform the surgeon during surgical planning, but also suggest the possible cognitive outcome and potential recovery. Preoperative findings can also be compared postoperatively to assess surgical results and to monitor cognitive functions during the follow-up. Nevertheless, only a few studies reported the tests included in the neuropsychological assessment together with the fMRI tasks for the preoperative planning.

Previous reviews focused on the multimodal MRI assessment of healthy brain aging and neurodegenerative diseases [30,31], but the literature focusing on patients with gliomas is scarce. In this context, motivated by the need for defined functional treatment protocols in brain tumor surgery, this review specifically targeted this patient population. The current study aimed to systematically review scientific evidence to identify studies including both the preoperative neuropsychological assessment and the fMRI task protocol, contributing to improving the neurosurgical treatment of gliomas.

## 2. Materials and Methods

Preferred Reporting Items for Systematic Reviews and Metanalyses (PRISMA) guidelines were followed for the systematic review [32,33]. A systematic search of the PubMed electronic database was conducted in February 2023 by cross-matching the following keywords: functional, magnetic resonance imaging, fMRI, MRI, brain tumor, glioma, task, neuropsy*. We included English studies published before February 2023. After duplicate removal, two researchers (MP and FB) independently reviewed titles and abstracts to identify articles of interest. Disagreement was resolved with a discussion that involved a third researcher (DC). We included studies that reported both neuropsychological tests and functional neuroimaging studies. Exclusion criteria included tumors not affecting the brain, secondary brain tumors, patients under 18 years old (given that cognitive results may be affected by developmental brain plasticity mechanisms), no preoperative assessment, resting state fMRI only, or healthy sample population/preclinical studies.

The articles were then evaluated, looking for correlations between neuropsychological assessment and fMRI task results, defined as the quantitative outcome. Papers reporting tests and tasks but without specifying the results attained by the patients in them, or exclusively showing the results of the postoperative assessment, were only included qualitatively according to the PRISMA guidelines (i.e., not meeting the review criteria but reporting additional beneficial results). The current review has been registered in the Open Science Framework (OSF) registry (https://doi.org/10.17605/OSF.IO/8DCZG; accessed on 10 June 2023).

The “Risk Of Bias In Systematic reviews” (ROBIS) assessment tool was employed to check for bias in the review process [34].

## 3. Results

**Figure 1:**
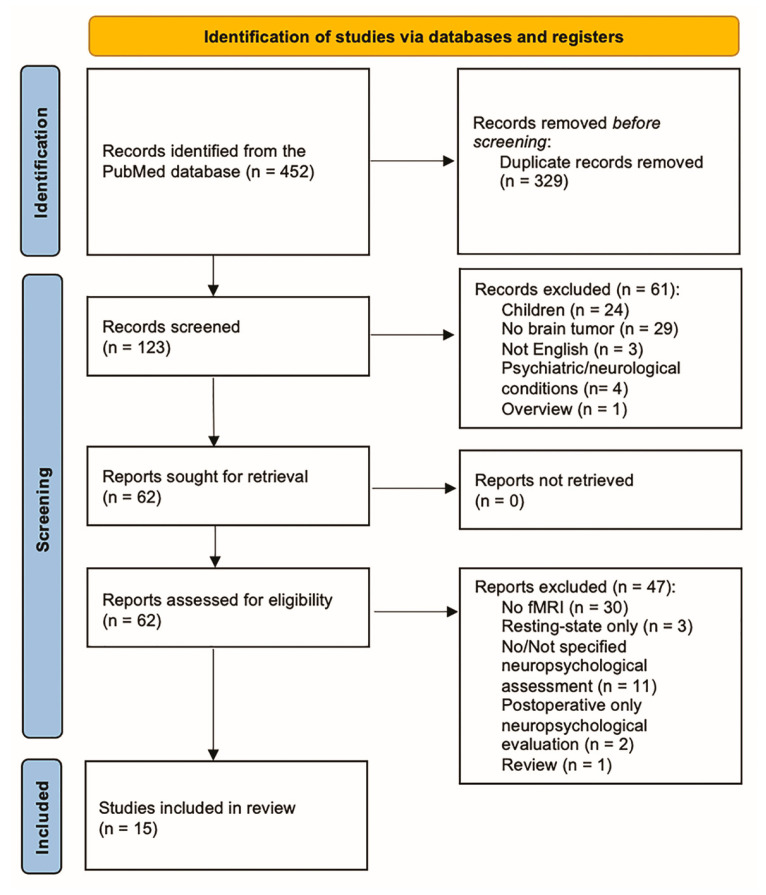
PRISMA flow diagram of the systematic review [33].

We identified 123 papers after duplicate removal. After title and abstract analysis, 62 articles were identified for full-text analysis. Eligibility evaluation led to the inclusion of 15 articles in the systematic review (Figure 1). According to the ROBIS assessment tool, we identified a low risk of bias in the “study eligibility criteria”, “identification and selection of studies”, and “data collection and study appraisal” domains. Based on the heterogeneity of results and the low number of eligible studies, a high risk of bias was identified in the “synthesis and findings” domain.

Nevertheless, we aimed to address all the concerns while interpreting the findings. We highlighted the relevance of each included study but avoided emphasizing results based on statistical significance. We also state the limitations of the current review in the paper’s discussion section.

Detailed results about the included papers and the employed neuropsychological and fMRI tasks are shown in Table 1 (we report specific fMRI tasks and neuropsychological tests extensively in Supplementary Materials). The aims of those studies were rather heterogeneous, as were the methods and conclusions. Only one study was published in 2006, reporting a single patient [35]. All the remaining papers were published after 2010: one in 2010 [36], one in 2011 [37], one in 2014 [38], one in 2015 [39], two in 2017 [40,41], two in 2019 [42,43], three in 2020 [44,45,46], and three in 2022 [47,48,49].

Their main results are discussed in the following sections according to the cognitive function investigated in the attempt to draw general conclusions about the clinical relevance of integrating pre-surgical neuropsychological assessment and fMRI. As a qualitative result, we found two more studies reporting only the details of the neuropsychological evaluation administered postoperatively [50,51].

### 3.1. Sensorimotor Functions

In the neurological context, the sensorimotor domain aims to integrate the sensory/perceptual component for processing stimuli and the motor response. Within this domain, four of the studies selected in this review reported the fMRI mapping of motor cortices in glioma patients (Table 2). 

Most of these studies focused on the general mapping of motor cortices. This result was usually obtained by employing standard and well-established experimental fMRI paradigms (e.g., finger tapping) [43], together with neuropsychological scores investigating motor skills and praxis.

Concerning the investigation of specific functions of the sensorimotor cortex, only two studies were found. The first one, by Argiris et al., suggested how the use of specific motor and sensory neuropsychological tests can be related to the tumor-affected hemisphere. This proposal underlined the concept of functional cerebral lateralization: gliomas located in the right hemisphere are more susceptible to impact visuospatial domains. Right-lateralized gliomas more frequently result in neglect conditions. Therefore, an accurate estimation of correlated neuropsychological visuospatial profile (i.e., visuospatial short-term memory, constructional apraxia, constructive ability, etc.) could help the pre-surgical planning by focusing first on the impairments and then on the related brain functional areas specific for this type of tumors. The same context applies to left-lateralized brain tumors, where the focus will be on linguistic functioning instead. By doing so, the authors showed a discrimination between tumor hemispheric localization in the performance of tasks previously considered unrelated to hemispheric lateralization, such as motor imagery processes [44]. Indeed, left tumor patients presenting a lesion near somatotopic hand representations performed significantly worse on the mental rotation hand fMRI task, correlating with motor-evoked potential (MEP) amplitudes in the upper limb motor region, and highlighting the involvement of the motor system in motor imagery processes [44].

The second study, by Zacharia et al., focused instead on investigating the role the cerebellum plays in cognitive, motor, and emotional functions, potentially acting during the development and refinement of internal models in motor and cognitive functions. Specifically regarding the motor domain, authors employed classic experimental designs to test motor activation (e.g., finger tapping, lip movement, etc.). They demonstrated that besides the presence of gliomas, the cerebellar activation patterns noted on functional MRI and cerebro-cerebellar connections remain intact [43].

Hence, regarding the sensorimotor functions, this novel review provides insights about (i) the importance of considering brain tumor lateralization for an accurate neuropsychological assessment, and shows that (ii) the cerebellar function and its associated cognitive performance seem to be preserved even in the presence of cortical gliomas.

### 3.2. Language

Language is the cognitive ability to communicate through a system of conventional and structured rules. Given its paramount importance, the linguistic function was the cognitive domain mainly investigated in the presurgical phases of treatment for glioma patients, with ten studies exploring the topic (Table 3). 

The results of studies focusing on language were highly heterogeneous, with several experimental paradigms and neurocognitive assessments exploited. However, some main findings can be drawn.

Firstly, a large part of the studies focused on investigating the relationship between these three components: areas belonging to the neoplastic formation, the neuropsychological assessment of linguistic performance, and fMRI mapping of the language network. This mainly ensured the mapping of anatomo-functional language-related relationships within the brain. Preoperatively, the cognitive impairment of linguistic performance was associated with neoplastic formations involving the ventral precentral gyrus and the arcuate fasciculus [37]. Despite this, conserved landmarks of functional *pars opercularis* were observed with task-fMRI in these patients, making the region highly relevant in presurgical planning [37].

Furthermore, gliomas involving the uncinate fasciculus or right temporal lobe also significantly impacted linguistic performance in denomination tasks [36] and speech perception [49], respectively. In this context, fMRI effectively described task response in different sub-components of the language domain, with high specificity. For example, in right superior temporal lobe gliomas, speech perception was characterized by a lower activation within the tumor site and enhanced activation of the contralateral hemisphere, which was reversed during speech production [49]. Additional evidence concerns left-lateralized tumor response during phonemic fluency tasks, which not only exhibited classic activation in left temporal and parietal regions, but included increased activity in frontal regions strongly correlated with the behavioral executive components of this linguistic skill [47].

Additionally, other authors highlighted aspects related to intra-operative language assessment and its relationship with presurgical evaluation. Indeed, Leote et al. focused on the intraoperative consequences of impaired presurgical cognitive performance. They described how presurgical cognitive deficits led to a decreased DCS duration and consequently to lower reliability of the methodology [45], which was also reflected by an ineffective fMRI mapping of the relevant cognitive functions [45].

Moreover, the compensatory capability of the unaffected brain areas was also recently studied postoperatively in the context of the linguistic domain. The functional recovery of language functions seemed to rely on changes in activation near the surgical resection (not in the contralateral hemisphere) [38]. However, these changes in the activation pattern were unrelated to functional variations of the performance following surgery, as measured with neuropsychological testing [38]. Lastly, the histology of the tumors could also play a role in language function preservation, with grading being one of the most impacting factors on cognitive recovery. Mitolo et al. reported that low-grade tumors showed higher rightward frontal operculum fMRI activations and, therefore, better cognitive performance in tests measuring general cognitive abilities, semantic fluency, verbal short-term memory, and executive functions [47].

Therefore, some main findings can be drawn: (i) the anatomo-functional brain correspondence of linguistic performance is not straightforward; (ii) presurgical fMRI can help detect eloquent areas and regions specific for linguistic sub-components otherwise disregarded; (iii) linguistic cognitive impairments must be taken into account for an efficient intra-surgical cortical mapping; (iv) language plasticity processes depend on tumor features and are related to complex diaschisis changes. Nevertheless, results about the language domain across different studies were discrepant, and more robust evidence could be provided only by considering longitudinal studies.

### 3.3. Executive Functions

The last category of cognitive function investigated regards executive skills. These include many high-cognitive abilities, including sustained and selective attention, response and inhibition control, working memory, and processing speed. They comprise the capabilities necessary for monitoring and controlling our behavior to reach a chosen goal. In this review, we selected six studies investigating the above-cited cognitive processes (Table 4).

The majority of the selected studies focused their investigations on working memory capacity by explicitly looking at changes in large-scale functional networking. Lang et al. showed that the task-evoked reconfiguration of the frontoparietal network (FPN; i.e., the executive network) correlates with cognitive performance, suggesting that its reconfiguration may play a role in cognitive deficits in brain tumor patients [41]. Nevertheless, a higher average connectivity within the FPN or in the parietal region of the tumor-affected hemisphere was associated with lower cognitive scores, and a lower connectivity of the parietal region of the non-tumor hemisphere was associated with worse neuropsychologic outcomes [41]. Therefore, patients with less connectivity in the FPN in the tumor hemisphere had preserved cognition. Alternatively, the authors hypothesized that the presence of the glioma may result in inefficient processing in the FPN due to maladaptive brain reorganization [41]. Furthermore, the fMRI results reveal normal central executive network (CEN) activation in glioma patients but a reduced default mode network (DMN) deactivation. This reduced responsiveness of the DMN may suggest that cognitive deficits reflect a reduced capacity to achieve a brain state necessary for normal cognitive performance, rather than the abnormal functioning of executive brain regions [46].

Additionally, one paper underlined the role of histology, showing that high-grade gliomas were significantly associated with lower cognitive flexibility and working memory capacity [42]. Lastly, tumor site was also reported as a relevant variable to consider pre-surgically: frontal tumors and left hemisphere lesions led to lower working memory skills [42].

On the other hand, Arbula et al., studying sustained attention and response control, concluded that right prefrontal damage led to frequent target omissions, probably due to sustained attention lapses, and that left prefrontal patients showed both target omissions and high false alarm rates to warning stimuli, suggesting a decisional rather than inhibitory impairment [40]. The anatomo-functional correlation of gliomas showed that left ventrolateral and dorsolateral prefrontal lesions were associated with target discrimination failure. In contrast, right ventrolateral and medial prefrontal lesions correlated with target detection failure [40].

One study focused instead on the self-monitoring function, with a particular interest in the functional role of the insular cortex. It proposed a new multimodal protocol combining DCS, awake surgery, and fMRI to measure self-monitoring skills with a modified version of the Stroop task. They reported differences in metacognitive domains of glioma patients, showing (i) increased difficulties in detecting action–outcome mismatches during insular DCS, and (ii) significant insular BOLD activations during outcome incongruences for self-made actions [48]. This highlights the importance of considering the insula activation in executive processing, especially in the metacognitive domain, for patients undergoing surgery.

Globally, these preliminary findings may imply that (i) executive function performance, such as working memory, is highly susceptible to plastic connectivity changes in related brain networking; (ii) such changes can relate to both attentive (FPN) and general (DMN) functional networks; (iii) tumor features, such as grade and site, can be negative predictors of executive performance; (iv) there is an existing correspondence between tumor site and attentive performance; (v) executive processing together with its networking can also be related to metacognitive skills.

### 3.4. Additional Studies (PRISMA Qualitative Analysis)

In addition to this review, two studies reporting postoperative-only neuropsychological assessment together with fMRI mapping within the above-cited cognitive domains were found and, therefore, were qualitatively included.

In greater detail, Amiez et al. studied the sensorimotor function and specifically the role of the rostral part of the left dorsal premotor cortex in four patients with low-grade tumors close to that region [50]. The fMRI task’s experimental design undertaken pre- and postoperatively was based on a visuomotor conditional task (i.e., the ability to select between competing responses based on previously defined conditional rules). The employment of the task enabled the localization of the functional activation area in the proximity of the tumor area in all four patients, enabling the exact delineation of premotor regions and aiding the planning of the surgery efficiently. This was further corroborated not only by an optimization of the EOR, but also by the absence of postoperative deficits in the visuomotor conditional task [50].

Quirarte et al. reported a case report of a left superior frontal glioma exhibiting linguistic impairments after surgery (i.e., language supplementary motor area syndrome). This study demonstrated the potential of fMRI mapping of the linguistic domain not only for localization purposes (i.e., surgical mapping), but also to gain better insights about plasticity processing occurring after surgery, and how these can be related to neuropsychological deficits exhibited by the patients [51].

## 4. Discussion

Function-preserving, maximally safe resection for brain tumors relies upon changes in the clinical approach. Indeed, not only does it consist of applying intraoperative mapping methods, but also improving the preoperative evaluation of patients and performing accurate presurgical planning to identify the relationship between the tumor and cortical and subcortical eloquent areas [15]. FMRI is a widely employed technology to identify the functional involvement of the cortical regions at rest or during different tasks [16,17,21]. The same tasks used during fMRI can be applied during awake surgery to obtain consistent results during direct cortical stimulation (DCS) [46]. Sensorimotor and language tasks are commonly used, but other functions (e.g., executive and attentive functions) are evaluated sporadically [40,41,42,46,48], and the task’s experimental design is not standardized among different centers [18,19,22,26]. Furthermore, integrating fMRI results with neuropsychological assessment increases the variability of protocols and reduces the comparability of the outcome [29].

In this review, we found only 15 studies reporting both the fMRI tasks and neuropsychological tests included in the preoperative evaluation of patients. Previous evidence provides insights into the following cognitive domains: (i) sensorimotor functions, which can be efficiently mapped during standard motor paradigms and conserved by pre-surgically investigating visuospatial abilities; (ii) language, which historically is the most largely mapped cognitive function in glioma patients, with the recent efficient brain localization of different linguistic sub-components and related improvements in the recovery of the linguistic function after surgery; (iii) executive abilities, which might depend on resilient plastic processes of large-scale networks’ connectivity, especially in the case of working memory. Nevertheless, the correlation between the results of the two examinations has yet to be extensively evaluated.

### 4.1. Anatomo-Functional Correlations of fMRI Mapping and Cognitive Performance

The first clinical implication of the investigated studies reporting fMRI data and neuropsychological assessment is the definition of correlations between areas with neoplastic involvement, functional brain sites, and cognitive status. These correlations may help in designing a specific multimodal presurgical planning strategy to improve the functional recovery of the patients. Currently, a broader part of the available literature focuses on language. But in this review, we demonstrated how, even for the same cognitive function (i.e., language), there is considerable variability across studies evaluating different eloquent areas as sub-parts of the cognitive network without a precise domain localization. Therefore, fMRI mapping, together with assessing neuropsychological correlates of a certain cognitive function, becomes influential if the tumor infiltrates one or more areas belonging to the supposed spatial localization of the corresponding neural network. In this context, language function is one of the most suitable candidates across cognitive domains, given the ease of its preoperative evaluation, intraoperative testing, and monitoring during follow-up [19,22]. On the contrary, evidence regarding other functions, such as sensorimotor, perceptual, and executive functions, is scarce and anecdotal. Therefore, more studies are needed to determine anatomo-functional correlations of cognitive domains at the single-subject level in glioma patients to precisely define a tailored surgical procedure.

### 4.2. Integration of fMRI Data and Neuropsychological Assessment

Secondly, only a few of the included studies emphasized the relationship between neuropsychological evaluation and fMRI tasks, reporting heterogeneous results. Some authors reported a complementary role of the two preoperative assessments; for example, regarding speech disturbances, fMRI-positive areas correlated with presurgical neuropsychological language tests were found in the frontal operculum [37,45], besides the well-known involvement of the arcuate fasciculus. Indeed, Schouwenaars et al. corroborated this hypothesis by showing how lower fMRI in-scanner performances in glioma patients compared to controls were associated with the same cognitive impairment during neuropsychological testing [46].

Further plastic evidence about specific compensatory and resilient fMRI activations, consequent to tumor presence, was given by Mitolo and colleagues, who assessed how they are positively correlated with a better cognitive performance in tests measuring general cognitive abilities (especially in semantic fluency, verbal short-term memory, and executive functions) [47]. Therefore, a correlation between fMRI and neuropsychological assessment is confirmed based on the currently available data. In cases where the two methods disagree, they should be seen as complementary tools, integrating their results for better surgical planning.

### 4.3. Role of fMRI-Positive Regions during Surgery

The most apparent transposition of functional data to surgical procedures is exhibited in the correlating of fMRI data and intraoperative monitoring with DCS to detect the spatial distribution of “function-positive” spots [45].

In this review, the only work reporting correspondence between DCS and fMRI was that by Leote, which was equal to 100% for the precentral gyrus for motor function and 84% regarding the opercular frontal inferior gyrus for language function. They found a correlation between worse presurgical neuropsychological performance and decreased DCS duration. Nevertheless, they stated that presurgical language disturbances limited the applicability of DCS mapping in awake surgery [45]. Indeed, according to the author’s interpretation, this was due to the surgeon’s decision to proceed with the tumor resection, having considered the systematic errors in language tasks instead of performing more efficient cortical mapping. Errors in language tasks without applying DCS were seen; therefore, the patients were engaged in spontaneous conversation, which demands a lower cognitive load but was not evaluated in fMRI paradigms [45].

Additionally, the surgeon focused more on negative cortical regions after DCS than positive fMRI regions, probably because of an unconscious preference for DCS for brain eloquent function mapping [45]. In addition to preoperative assessments with fMRI and comparisons with DCS results, some papers suggested the direct implementation of functional data intraoperatively [35,45,50]. This process can be done by fusing fMRI data into morphological MRI sequences and employing intraoperative navigation. Several pieces of evidence have highlighted how the intraoperative use of fMRI data in the context of neuronavigation can add highly informative and integrated knowledge about tumor features during resection [19]. Indeed, neuronavigation with fMRI increases both the neurosurgeon’s accuracy and the identification of target regions for resection. Furthermore, information can be combined with neuronavigation in a multimodal manner, taking advantage also of the white matter fiber-tracking visualization [52,53]. The superimposition of white matter tracts on eloquent functional brain areas during neuronavigation may support both the evaluation of better functional limits for the resection and intraoperative neurophysiological mapping. As noted by Leote et al., the usage of navigated fMRI data during the surgery seems to not influence or improve DCS in practice [42], and therefore, further multimodal studies are still needed.

Finally, as a potential future development, Argiris et al. reported that patients performing worse on the mental rotation hand fMRI task had lower MEP amplitudes in the upper limb motor region during transcranial magnetic stimulation [44]; this may also have an impact on DCS during surgery if tested systematically.

### 4.4. Patient-Tailored Protocols According to the Lesion Site and Plasticity Evaluation

Different authors suggested that selecting well-adjusted and individualized neuropsychological tasks pre- and intraoperatively may enable the development of personalized and more efficient brain mapping protocols [39]. Yamamoto et al. pushed this concept forward, suggesting that fMRI findings, also regarding brain plasticity, may have important implications for the surgical management of patients with brain tumors [49]. Kamada et al. highlighted the same evidence, identifying how fMRI can successfully map the dissociation of cognitive functions, particularly within the language domain. Indeed, they probed that fMRI activation during abstract/concrete categorization tasks was located within their patients’ right superior temporal region, in contrast to the activation of the left superior temporal and left supramarginal gyri in controls [35]. Moreover, knowledge of the areas showing functional reorganization may help avoid unintentional damage during neurosurgery [49]. Future prospective studies are needed to define fMRI and neuropsychological protocols specific to tumor locations and types, which will help clinicians select the most appropriate assessment method for the individual patient. In this context, preoperative task-based fMRI is a feasible and highly sensitive tool for localizing eloquent cortical areas in patients affected by brain tumors. Nevertheless, its prognostic role, regarding reduced morbidity and improved oncologic outcome, still needs to be definitively addressed and clarified.

### 4.5. Limitations of the Current Study

The major limitation of the current study is the limited number of studies that met the inclusion criteria. This paucity reflects the heterogeneity of the literature regarding fMRI studies, behavioral experimental design, neuropsychological tests, and specific aims investigated.

This systematic review included only brain gliomas, discarding secondary nervous system tumors. Here, we did not specifically focus on precise brain tumor sites and grading, given our aim of including the vast majority of relevant neuro-oncologic works in the field. Nevertheless, this contributed to a lack of straightforward conclusions; findings from the literature underlined this complexity. They were characterized by high variability in concordance rates, with sensitivity and specificity ranging from 59 to 100% and 0 to 97% [54,55]. Moreover, the difficulty in the generalizability of the results not only relates the hypothesis of each study, but is also caused by the nature of neural network connections related to cognitive functions, which are highly complex and require several heterogeneous tasks and tests in extensively mapping them. In addition, diverse acquisition protocols and image post-processing techniques may also significantly impact the areas identified as related to a specific cognitive function in fMRI studies. The heterogeneity of data further dilutes the strength of any current recommendations made in the review.

### 4.6. Future Perspectives

Based on the results of this systematic review, further studies focusing on the relationship between the preoperative mapping of eloquent brain areas and neuropsychological profiles in gliomas are needed. Attention should be primarily set on defining acquisition protocols that can be tailored to single patients, but assessing standardized and reproducible parameters, ideally including:

- Preoperative and postoperative evaluations, as longitudinal comparisons have not been made extensively in the previous literature;
- fMRI tasks focused on specific cognitive functions and put in perspective with relative neuropsychological assessments, also taking into account the tumor site and hemisphere;
- Correlations of specific neuropsychological tests with experimental fMRI tasks’ results to identify clinical criteria for the indication of preoperative fMRI.

## 5. Conclusions

The results of this review re quite heterogeneous, and only a few papers satisfied the inclusion criteria, significantly impacting the generalizability of the findings. Most studies considered language as the most commonly evaluated cognitive function in clinical practice. In the literature, fMRI and neuropsychological assessments differed in almost every paper regarding the studied functions, but also the protocols fused to assess a specific function, impacting the chance of drawing broad conclusions and protocol suggestions. Furthermore, the fMRI and neuropsychological results demonstrated high variability even when evaluating the same cognitive domain [37]. Nevertheless, the correlation between neuropsychological and fMRI results reported by some authors suggests that altered functions during the neuropsychological assessment may help identify patients who could benefit from an fMRI evaluation in general and, possibly, which specific function should be tested [45]. However, this should follow a decision based also on tumor site and suspected grading, as low-grade tumors seem to be associated with higher levels of plasticity processes and better cognitive functions [47]. Based on the current literature, the neuropsychological evaluation and fMRI complement each other in preoperative patient assessment, surgical planning, and within the surgical procedure itself through neuronavigation and awake testing. They could potentially even improve post-surgical outcomes in the future. However, clinicians must consider multiple patient-related and tumor factors to determine the most appropriate protocol.

## Figures and Tables

**Table 1:** Results of the systematic review.

Author	Patients (N)	WHO Grade	Hemisphere and Location			Surgery	Cognitive Function Domain	fMRI Tasks	Neuropsychological Tests	Awake Surgery Assessment
			L	R	NA/Both					
[40]	25	18 HGG 7 LGG	7 prefrontal HGG 1 prefrontal LGG	4 prefrontal HGG 3 prefrontal LGG	7 non-prefrontal HGG 3 non-prefrontal LGG	Y	Executive	Go/No-Go task	MMSE, IQ, Verbal short-term memory and working memory Selective and divided attention visuospatial short-term memory Phonemic fluency	N
[44]	20	13 HGG 7 LGG	2 premotor HGG 4 motor HGG 2 sensorimotor HGG 1 parasagittal HGG 2 premotor LGG 2 motor LGG 1 L sensorimotor LGG	1 premotor HGG 2 motor HGG 1 sensorimotor HGG 1 premotor LGG 1 motor LGG	-	N	Sensorimotor Language	Motor localizer tasks, general motor imagery ability, conceptual knowledge of actions, lexical grammar processing, verb naming	Nonverbal intelligence Visuospatial short-term memory Constructional apraxia Visuospatial/constructive ability and planning Attentional neglect Visuoconceptual and visuomotor tracking Verbal short-term memory Buccofacial and ideomotor apraxia Noun naming and phonemic fluency	N
[37]	19	13 HGG 6 LGG	ventrolateral frontal (anterior and posterior groups)	-	-	N	Language	Verb generation task, orofacial apraxia	Phonemic fluency Semantic fluency Orofacial apraxia	N
[42]	26	13 HGG 13 LGG	8 HGG 7 LGG	5 HGG 6 LGG	4 of the HGG were frontal 10 of the LGG were frontal (Side NA)	N	Executive	N-back task	Cognitive flexibility (shifting attention)	N
[48]	1	II	1 fronto-insular	-	-	Y	Executive	Stroop task	Executive functions and attention Working memory Inhibition Mental flexibility Set shifting abilities Verbal fluency (semantic and phonological) Language production and naming Verbal comprehension Insular-related functioning (Empathy scale and emotion recognition) Mood	Y
[35]	1	II	-	1 insular	-	Y	Language	Verb generation task, abstract/concrete categorization	Language functions IQ Memory Visual retention	N
[38]	20	3 HGG 15 LGG	6 frontal 1 fronto-temporal 1 parietal 1 fronto-parietal 2 temporo-occipital 1 temporo-parietal 2 temporal	4 frontal 1 fronto-temporal 1 parietal	-	Y	Language	Verb generation task	IQ Abstract reasoning Cognitive processing speed Executive functioning Attention Working memory	Y
[41]	16	11 HGG 5 LGG	8 frontal 2 insular 1 temporal 1 frontoparietal	2 frontal 1 temporoparietal 1 temporal	-	Y	Executive Sensorimotor Language	N-back task, motor and language tasks	National Institutes of Health Cognitive Battery	N
[45]	18	11 HGG 7 LGG	10 frontal 5 temporal 1 insular 2 parietal	-	-	Y	Language Sensorimotor	Verb generation, semantic and syntactic decision tasks, motor tasks	Picture-naming Nonverbal visual semantic decision Verb-generation task	Y
[47]	15	10 HGG 5 LGG	10 frontal	5 frontal	-	Y	Language	Phonemic fluency task	MMSE, IQ Naming Phonemic verbal fluency Category fluency Short-term verbal memory and episodic memory Visuospatial short-term memory span and long-term visuospatial memory Visuoconstructive and planning abilities Attention and executive functions Depression and anxiety Cognitive reserve	Y
[36]	44	19 HGG 25 LGG	frontal and temporal	-	-	Y	Language	Word generation, picture naming tasks	Non-verbal intelligence Verbal and visuospatial, short- and long-term memory Selective and divided attention Orofacial, ideomotor, and constructional apraxia Spatial cognition Phonemic and semantic fluency Naming tasks Sentence comprehension Repetition	Y
[46]	46	25 HGG 21 LGG	13 HGG 10 LGG (possible involvement of central executive network or default mode network)	12 HGG 10 LGG (possible involvement of central executive network or default mode network)	1 LGG both	N	Executive	N-back task	Cognitive performance	N
[39]	1	III	premotor	-	-	Y	Language	Verb generation task	SMA functions Picture description Semantic and phonological verbal fluency Motor execution Processing speed Working memory Verbs and nouns generation	Y
[49]	19	HGG and LGG	-	3 posterosuperior temporal lobe	16 supratentorial (controls, NA)	Y	Language	Speech perception, object recognition, auditory short-term memory holding	Behavioral testing with language-related and cognitive non-language tasks	N
[43]	23	NA	-	-	23	N	Sensorimotor Language Executive	Sensorimotor processing, language, working memory, executive function, visual function, auditory function	MoCa	N

Legend: HGG, High-grade glioma. IQ, Intelligence quotient. LGG, Low-grade glioma. MMSE, Mini mental state examination. N, No. NA, Not available. WHO, World Health Organization. Y, Yes. MoCa, Montreal Cognitive Assessment.

**Table 2:** Selected studies investigating presurgical sensorimotor functions.

Author	Patients (N)	WHO Grade	Surgery	fMRI Tasks	fMRI Measures	Neuropsychological Tests (Related to Task)	Main Results	Awake Surgery	Task during Awake Surgery
[44]	20	13 HGG 7 LGG	N	Motor localizer tasks General motor imagery ability Conceptual knowledge of actions	Somatotopic cortical mapping (mouth, hand and feet) Imagery questions (joint movement, hands spatial position during action production) Mental rotation task Kissing and Dancing Test	Visuospatial short-term memory Constructional apraxia Visuospatial/constructive ability and planning Attentional neglect Visuoconceptual and visuomotor tracking Buccofacial and ideomotor apraxia	Involvement of the motor system in motor imagery processes	N	
[41]	16	11 HGG 5 LGG	Y	Motor tasks		NA	FPN functional connectivity is related to cognitive outcomes after surgery	Y	NA
[45]	18	11 HGG 7 LGG	Y	Motor tasks	NA	Picture-naming Nonverbal visual semantic decision task Verb-generation task	Navigated fMRI data did not influence DCS in practice	Y	Picture naming, nonverbal visual semantic decision task
[43]	23	NA	N	Sensorimotor processing	Finger tapping Toe movement Lip movement	MoCa (Montreal Cognitive Assessment)	Simultaneous cerebellar activation across different cognitive domains (except visual)	N	

Legend: HGG, High-grade glioma. IQ, Intelligence quotient. LGG, Low-grade glioma. MMSE, Mini-mental state examination. N, No. NA, Not available. WHO, World Health Organization. Y, Yes. DCS, direct cortical stimulation. FPN, Frontoparietal network.

**Table 3:** Selected studies investigating presurgical linguistic functions.

Author	Patients (N)	WHO Grade	Surgery	fMRI Tasks	fMRI Measures	Neuropsychological Tests (Related to Task)	Main Results	Awake Surgery	Task during Awake Surgery
[44]	20	13 HGG 7 LGG	N	Lexical grammar processing Verb naming	Verbs conjugation discrimination Verb oral naming task from BADA	Nonverbal intelligence Noun naming Phonemic fluency	Lexico-semantic processing of action not compromised by sensorimotor area lesion	N	
[37]	19	13 HGG 6 LGG	N	Verb generation task	Verb generation	Phonemic fluency Semantic fluency Orofacial apraxia	Functional activation of pars opercularis	N	
[35]	1	II	Y	Verb generation task Abstract/concrete categorization	Silent verb generation related to a noun Categorization of a word	Language functions IQ Memory Visual Retention	Activation of left frontal regions	N	
[38]	20	3 HGG 15 LGG	Y	Verb generation task	Covert articulation of a verb related to a noun	IQ	Perilesional functional reorganization of language areas	Y	Motor and language tasks
[45]	18	11 HGG 7 LGG	Y	Verb generation task Semantic and syntactic decision tasks	Silent verb generation related to a noun Judgment of the semantic correctness of phrases	Picture-naming Nonverbal visual semantic decision Verb-generation task	DCS duration is not reduced by the use of fMRI mapping	Y	Picture naming Nonverbal visual semantic decision task
[47]	15	10 HGG 5 LGG	Y	Phonemic fluency task	Covert generation of a noun starting with a given letter	MMSE, IQ Naming Phonemic verbal fluency Category fluency Short-term verbal memory and episodic memory	Left hemispheric dominance in temporal and parietal regions	Y	Specific language tests
[36]	44	19 HGG 25 LGG	Y	Word generation Picture naming tasks	Language dominance	Non-verbal intelligence Orofacial, ideomotor, and constructional apraxia Phonemic and semantic fluency Naming tasks Sentence comprehension Repetition	Role of the uncinate fasciculus in the retrieval of a word form for proper names	Y	Language with blocks of items (living, non-living, faces, verbs)
[39]	1	III	Y	Verb generation task	Covert generation of a verb starting with a given noun	Picture description Semantic and phonological verbal fluency Verbs and nouns generation	No functional change post-surgically in the verb generation task	Y	Language tasks (ability to repeat words and non-words and to generate verbs)
[49]	19	HGG and LGG	Y	Speech perception Object recognition Auditory short-term memory holding	Recognition of the semantic relationship	Behavioral testing with language-related and cognitive non-language tasks	Importance of right temporal lobe for language processing	N	
[43]	23	NA	N	Language processing	Word generation Verb generation Sentence completion	MoCa	Simultaneous cerebellar activation across different cognitive domains (except visual)	N	

Legend: HGG, High-grade glioma. IQ, Intelligence quotient. LGG, Low-grade glioma. MMSE, Mini mental state examination. N, No. NA, Not available. WHO, World Health Organization. Y, Yes. MoCa, Montreal Cognitive Assessment.

**Table 4:** Selected studies investigating presurgical executive functions.

Author	Patients (N)	WHO Grade	Surgery	fMRI Tasks	fMRI Measures	Neuropsychological Tests (Related to Task)	Main Results	Awake Surgery	Task during Awake Surgery
[40]	25	18 HGG 7 LGG	Y	Go/No-Go task	Omissions and false alarms	MMSE, IQ, Verbal short-term memory and working memory Selective and divided attention Visuospatial short-term memory	Prefrontal areas underlie broader cognitive control processes (response selection, target detection)	N	
[42]	26	13 HGG 13 LGG	N	N-back task	2-back congruent conditions	Attention shifting	FPN plastic capacity plays a role in cognitive deficits	N	
[48]	1	II	Y	Stroop task	Informative feedback blocks	Executive functions Attention Working memory Inhibition Mental flexibility Set shifting abilities Insular-related functioning (empathy scale and emotion recognition)	Role of the insula in self-monitoring	Y	Awake mapping multimodal protocol (modified version of the Stroop task)
[41]	16	11 HGG 5 LGG	Y	N-back task	Difference between 0-back and 2-back congruent conditions	National Institutes of Health Cognitive Battery	FPN connectivity is associated with cognitive performance	N	
[46]	46	25 HGG 21 LGG	N	N-back task	Difference between 0-back and 2-back congruent conditions	Cognitive performance	Cognitive deficits associated with reduced DMN	N	
[43]	23	NA	N	Working memory, executive function	N-back task	MoCa	Simultaneous cerebellar activation across different cognitive domains (except visual)	N	

Legend: HGG, High-grade glioma. IQ, Intelligence quotient. LGG, Low-grade glioma. MMSE, Mini mental state examination. N, No. NA, Not available. WHO, World Health Organization. Y, Yes. MoCa, Montreal Cognitive Assessment.

## Data Availability

Data are contained within the article.

## Supplementary Materials

The following supporting information can be downloaded at: https://www.mdpi.com/article/10.3390/brainsci13101380/s1.

## Abbreviations

BOLD, blood oxygen level-dependent; CEN, central executive network; DCS, direct cortical stimulation; DMN, default mode network; EOR, extent of resection; fMRI, functional magnetic resonance imaging; GTR, FPN, fronto-parietal network, gross total resection; HGG, high-grade glioma; IQ, intelligence quotient; LGG, low-grade glioma; MEP, motor evoked potential; MMSE, mini mental state examination; OSF, open science framework; PRISMA, Preferred Reporting Items for Systematic Reviews and Metanalyses; ROBIS, risk of bias in systematic reviews; WHO, World Health Organization.
