# Supplementary material for: Neuropsychological Evaluation and Functional Magnetic Resonance Imaging Tasks in the Preoperative Assessment of Patients with Brain Tumors: A Systematic Review
Source: Brain Sci. 2023 Sep 28;13(10):1380. doi: 10.3390/brainsci13101380 (PMC10605177; doi:10.3390/brainsci13101380)
Supplement: Supplementary file 1 [file brainsci-13-01380-s001.zip › brainsci-2601987-supplementary.pdf]

Supplementary Materials

Author	Year	N. of Patients	WHO Grade	Side and Location			Surgery	fMRI tasks	Neuropsychological tests	Awake surgery assessment
				L	R	NA/Both				
Arbula S.	2017	37 (25 gliomas)	18 HGG<br>7 LGG	7 prefrontal HGG<br>1prefrontal LGG	4 prefrontal HGG<br>3 prefrontal LGG	7 non-prefrontal HGG<br>3 non-prefrontal LGG	Y	Prefrontal functions<br>Foreperiod task (2 cm×2 cm 'XX') displayed at the center of the screen with an auditory warning stimulus<br>Go/No-Go task (Two uppercase letters (A and E)	MMSE, IQ, Digit span, Trail making test (TMT) A-B, Corsi block span, Phonemic fluency	N
Argiris G.	2020	20	13 HGG<br>7 LGG	2 premotor HGG<br>4 motor HGG<br>2 sensorimotor HGG<br>1 parasagittal HGG<br>2 premotor LGG<br>2 motor LGG<br>1 L sensorimotor LGG	1 premotor HGG<br>2 motor HGG<br>1 sensorimotor HGG<br>1 premotor LGG<br>1 motor LGG	-	N	Sensorimotor cortex<br>Motor localizer tasks for somatotopic cortical mapping with mouth, hand, and feet movements<br>General motor imagery ability (Italian version of the Florida Praxis Imagery Questionnaire: answer imagery questions about joint movement or the spatial position of the hands during action production)<br>General motor imagery ability (mental rotation task with rotated images of hands and feet - decide if representing the right or left)<br>Conceptual knowledge of actions (written verb subtest of the Kissing and Dancing Test: probe word and choosing of the corresponding verb)<br>Lexical grammar processing (subset of Italian verbs relating to the hand, face, and foot in the first- or third-person singular or plural, asking to decide if the word represents a third-person singular form)<br>Verb naming (verb oral naming task from the BADA)	Nonverbal intelligence (Raven's Colored Progressive Matrices test)<br>Visuospatial short-term memory (Corsi block span, forward and backward)<br>Constructional apraxia (Figure drawing)<br>Visuospatial/constructive ability and planning (Clock-drawing test)<br>Attentional neglect (Behavioral Inattention Test)<br>Visuoconceptual and visuomotor tracking (TMT)<br>Verbal short-term memory (Digit span, forward and backward)<br>Buccofacial and ideomotor apraxia, with language comprehension (Token test)<br>Noun naming (subsection of the BADA)<br>Phonological fluency	N
Bizzi A.	2011	19	13 HGG<br>6 LGG	ventrolateral frontal (anterior and posterior groups)	-	-	N	Language and Orofacial apraxia<br>Verb generation task (block design with nine 24-sec alternating periods and two conditions was used. In the experimental condition patients were asked to generate a verb in response of every noun visually presented)	Language (AAT)<br>Phonemic fluency (Produce as many words as possible beginning with the letters "F", "P" and "L" in three trials lasting 1 minute)<br>Semantic fluency (Produce as many words as possible for categories "clothes", "animals" and "fruits" in three trials lasting 1 minute)<br>Orofacial apraxia (Imitation of voluntary, non-speech oral movements)	N
De Baene W.	2019	26	13 HGG<br>13 LGG	8 HGG<br>7 LGG	5 HGG<br>6 LGG	4 of the HGG were frontal<br>10 of the LGG were frontal<br>(Side NA)	N	Cognitive flexibility<br>N-back task, part of a larger fMRI experiment with varying working memory load.<br>Task presented in blocks of 30 s, with two or three consecutive conditions (instructions 4 seconds earlier), interleaved with rest blocks of 15 s: attention to a fast sequence of consonants and response if a stimulus was equal to one presented 2 trials before (pushing a button with the right hand)	Cognitive flexibility (Shifting attention task that is part of the CNS VS)	N
Gomez-Andres A.	2022	1	II	1 fronto-insular	-	-	Y	Insular self-monitoring<br>The Stroop experimental paradigm (adapted: The target stimulus appeared for 500 msec, which varied in terms of the laterality-color congruency and word-color congruency variables (8 trial types in total) The patient was instructed to respond as fast as possible to the ink color ignoring the word by pressing either left (blue) or right (red)).	Executive functions such as attention (Digits-direct from Barcelona-Revisado test, TMT-part A)<br>Working memory (Digits-inverse from Barcelona-Revisado test), "Letters and Numbers" and "Arithmetic" subtests from the Wechsler Adult Intelligence Scale-IV,<br>Inhibition (Stroop Task), Mental flexibility (TMT-part B), Set shifting abilities (Wisconsin	Y<br>Awake mapping multimodal protocol (neuropsychological assessment, fMRI and DCS) to assess anterior Insular Cortex-related functions

									Card Sorting Test), Verbal fluency (semantic and phonological)<br>Language production and naming (Boston Naming Test), Verbal comprehension (Token Test)<br>Insular-related functioning (Empathy scale with Interpersonal Reactivity Index, Emotion recognition test with Ekman 60 faces Test)<br>Mood (Hamilton Anxiety Rating Scale, Hamilton Depression Rating Scale)	(Modified version of the Stroop task)
Kamada k.	2006	1	II	-	1 insular	-	Y	Language<br>Verb generation task (Generate silently a verb related to each noun presented)<br>Abstract/concrete categorization task (Categorize the presented word covertly as abstract or concrete)	Western Aphasia Battery, Wechsler Adult Intelligence Scale–Revised, Wechsler memory test, Benton Visual Retention Test	N
Kristo G.	2014	20	3 HGG<br>15 LGG	6 frontal<br>1 fronto-temporal<br>1 parietal<br>1 fronto-parietal<br>2 temporo-occipital<br>1 temporo-parietal<br>2 temporal	4 frontal<br>1 fronto-temporal<br>1 parietal	-	Y	Language<br>Verb generation task (Language blocks: noun presented on the screen every 3 sec to sub-vocally (covert articulation) generate a related verb for the presented noun, alternated with non-language control blocks)	IQ (verbal task, National Adult Reading Test), Abstract reasoning (Raven's Advanced Progressive Matrices), Cognitive processing speed (TMT-part A), Executive functioning (TMT-part B), Attention span (Digit span forward), Working memory (Digit span backward)	Y<br>Motor and language tasks; incidentally, other tasks were used (e.g., counting and subtracting)
Lang S.	2017	16	11 HGG<br>5 LGG	8 frontal<br>2 insular<br>1 temporal<br>1 frontoparietal	2 frontal<br>1 temporo-parietal<br>1 temporal	-	Y	Cognitive flexibility<br>Motor and language tasks<br>N-back task (identify regions within the frontoparietal network: 1 run of four blocks of 2-back trials and five blocks of a 0-back control trial in an alternating fashion)	National Institutes of Health Toolbox Cognitive Battery 7 tasks: Dimensional Change Card Sort, Flanker Inhibitory Control and Attention, Picture Sequence Memory, Picture Vocabulary, Oral Reading Recognition, Pattern Comparison Processing Speed, and List Sorting Working Memory	N
Leote J.	2020	18	11 HGG<br>7 LGG	10 frontal<br>5 temporal<br>1 insular<br>2 parietal	-	-	Y	Motor and language tasks (verb generation, semantic and syntactic decision tasks)	Language (Picture-naming task, Nonverbal visual semantic decision task using a pyramid and palm tree test, verb-generation task)	Y<br>Picture naming and Nonverbal visual semantic decision task (same pictures shown in presurgical evaluation)
Mitolo M.	2022	15	10 HGG<br>5 LGG	10 frontal	5 frontal	-	Y	Language<br>Phonemic fluency task with a block design	MMSE, General intelligence (non-verbal Raven's Colored Progressive Matrices)<br>Language skills (The Boston Naming Test, Phonemic Verbal Fluency Test, Category Fluency Test)<br>Short-term verbal memory and episodic memory (Digit Span forward, Immediate and delayed recall of Babcock Story Recall Test)<br>Visuo-spatial short-term memory span and visuo-spatial long-term memory (Corsi's Block Test, Delayed recall of the ROCF)<br>Visuo-constructive and planning abilities (Copy of ROCF) Attention and executive functions (Frontal Assessment Battery, Stroop test, Digit TMT A and B)<br>Depression and anxiety (Beck Depression Inventory, State-Trait Anxiety Inventory)<br>Cognitive Reserve Index, SF-36	Y<br>Specific language tests (Boston Naming Test and selected items of the AAT chosen case-by-case)
Papagno C.	2010	44	19 HGG<br>25 LGG	frontal and temporal	-	-	Y	Language<br>Word generation and picture naming tasks	Non-verbal intelligence, Verbal and Visuospatial, short- and long-term memory	Y<br>Blocks of items (living, non-living,

									(Digit span, Corsi span, Word list learning, Supraspan learning, Rey figure reproduction)<br>Selective (Attentional matrices) and Divided attention (TMT)<br>Orofacial, ideomotor, and constructional apraxia<br>Spatial cognition<br>Language (Verbal fluency on phonological and semantic cue, Naming of famous faces, Picture naming of objects and actions, Naming by description, Pointing to picture, Sentence comprehension with Token Test and Picture-to-sentence matching task, Repetition of syllables, words, non-words and sentences)	faces, verbs) presented counterbalanced across patients; all the responses in the subsequent runs were reported
Schouwenaars I.	2020	46	25 HGG<br>21 LGG	13 HGG<br>10 LGG<br>(possible involvement of central executive network or default mode network)	12 HGG<br>10 LGG<br>(possible involvement of central executive network or default mode network)	1 LGG both	N	Cognitive flexibility<br>N-back task (a 2-back working memory task) and a baseline task to exclude activation associated with motor and visual processes: sequence of consonants that was presented in the center of the screen. For baseline, response to a target consonant; for 2-back, response if a stimulus was equal to a stimulus that was presented 2 trials before	Cognitive performance (Central Nervous System Vital Signs, 7 tests in this battery: verbal memory, visual memory, symbol digit coding, finger-tapping, Stroop III, shifting attention, and a continuous performance test)	N
Sierpowska J.	2015	1	III	premotor	-	-	Y	Language<br>Noun-based verb generation task (listening to an auditorily presented list of nouns, covertly name a relevant verb based on the name of the object)<br>Verb generation task (standard block design task)	SMA functions (Informative content of language from the Barcelona test), Picture description (Cookie Theft from the Boston Naming test), Semantic (animals) and phonological (letters: p, m, r) verbal fluency, Motor execution (Purdue Pegboard test), Processing speed-Wechsler Adult Intelligence Scale (digit-symbol coding and symbol search) and working memory- Wechsler Adult Intelligence Scale (Digit span, letter-number sequencing and arithmetic), Verbs and nouns generation (Image-based verbs generation task and noun-based verb generation)	Y<br>Language tasks (ability to repeat words and non-words, and to generate verbs)
Yamamoto A.	2022	19	HGG and LGG	-	3 posterosuperior temporal lobe	16 supratentorial (controls, NA)	Y	Language<br>Listening to pairs of spoken objects semantically related or unrelated (speech perception, object recognition, auditory short-term memory holding)<br>Aloud object naming in each of a series of pictures, linked with "and"<br>Control condition: seeing pictures of two objects (as in task 2) and semantically matching them (as in task 1)	behavioural testing performed by a speech and language therapist using the CAT (Swinburn et al., 2004) which consists of twenty one language related tests and six cognitive non-language tasks	N
Zacharia T.	2019	23	NA	-	-	23	N	Cerebellar activation<br>Sensorimotor processing (Finger tapping, Toe movement, Lip movement)<br>Language (Word generation, Verb generation, Sentence completion)<br>Working memory, Executive function (N-back task)<br>Visual function<br>Auditory function	Naming, Visuospatial executive function, Memory, Attention, Language, Abstraction, Delayed recall, Orientation (MOCA)	N

Legend: AAT, "Aachener Aphasie test"; BADA, "Batteria per l'Analisi dei Deficit Afasici"; CAT, Comprehensive aphasia test; CNS VC, Central Nervous System Vital Signs; DCS, Direct cortical stimulation; fMRI, Functional magnetic resonance imaging; HGG, High-grade glioma; IQ, Intelligence quotient; LGG, Low-grade glioma; MOCA, Montreal cognitive assessment; MMSE, Mini mental state examination; N, NO; NA, Not available; ROCF, Rey–Osterrieth complex figure; SF-36, Short Form Health Survey 36; SMA, supplementary motor area; TMT, Trail making test; WHO, World Health Organization; Y, Yes.
